# Unexpected synthesis and crystal structure of *N*-{2-[2-(2-acetyl­ethen­yl)phen­oxy]eth­yl}-*N*-ethenyl-4-methyl­benzene­sulfonamide

**DOI:** 10.1107/S2056989020015194

**Published:** 2020-11-20

**Authors:** Ayalew W. Temesgen, Minh Duc Luong, Hong Hieu Truong, Van Tuyen Nguyen, Thi Tuyet Anh Dang, Tuan Anh Le, Alexander G. Tskhovrebov, Victor N. Khrustalev

**Affiliations:** a Department of Chemistry, College of Natural and Computational Sciences, University of Gondar, 196 Gondar, Ethiopia; b Faculty of Chemistry, VNU University of Science, Vietnam National University, Hanoi, 334 Nguyen Trai, Hanoi, 100000, Vietnam; c Faculty of Science, Peoples’ Friendship University of Russia (RUDN University), 6 Miklukho-Maklaya, Moscow, 117198, Russian Federation; d Institute of Chemistry, Vietnam Academy of Science and Technology, 18 Hoang Quoc, Viet, Hanoi, Vietnam; e N.N. Semenov Federal Research Center for Chemical Physics, Russian Academy of, Sciences, Kosygina 4, Moscow, Russian Federation; f N.D. Zelinsky Institute of Organic Chemistry, Russian Academy of Sciences, 47 Leninsky Prosp., Moscow 119991, Russian Federation

**Keywords:** crystal structure, Petrenko–Kristchenko reaction, enamine, *PASS* software

## Abstract

The title compound, C_21_H_23_NO_4_S, obtained by alkaline treatment of 1,5-bis­(1-phen­oxy)-3-aza­pentane at moderate heating, is a *N*-tosyl­ated secondary vinyl­amine. An intra­molecular S=O⋯H—C hydrogen bond generates a 13-membered ring. The benzalacetone moiety adopts a *trans* conformation with respect to the C=C double bond, which is slightly longer than usual due to the conjugation with a neighbouring acetyl group. Theoretical predictions of potential biological activities were performed, suggesting that the title compound can inhibit gluconate 2-de­hydrogenase (85% probability), as well as to act as a mucomembranous protector (73%).

## Abstract 2

The title *N*-tosyl­ated secondary vinyl­amine was obtained by alkaline treatment of 1,5-bis­(1-phen­oxy)-3-aza­pentane at moderate heating. Theoretical predictions suggest that the title compound could inhibit gluconate 2-de­hydrogenase (85% probability), as well as to act as a mucomembranous protector (73%).

## Chemical context   

In our previous publications, we have reported the synthesis of new aza-crown ethers containing various fragments: γ-piperidone *via* the Petrenko–Kritschenko reaction (Levov *et al.*, 2006*a* ▸,*b* ▸, 2008 ▸; Anh *et al.*, 2012 ▸; Hieu *et al.*, 2016 ▸, 2019 ▸; Nguyen *et al.*, 2017 ▸; Dao *et al.*, 2019 ▸), diazine (Hieu *et al.*, 2012 ▸, 2013 ▸), or triazine (Hieu *et al.*, 2009 ▸, 2012 ▸; Khieu *et al.*, 2011 ▸). Among them, several obtained aza­crown ethers exhibited cytotoxicity to human cancer cell lines: *Hepatocellular carcinoma* (Hep-G2), *Human lung adenocarcinoma* (Lu1), *Rhabdosarcoma* (RD), *Human breast adenocarcinoma* (MCF-7) (Dao *et al.*, 2019 ▸; Anh *et al.*, 2019 ▸). For further syntheses of new aza-crown derivatives, a modification of multi-component condensation reactions based on the Petrenko–Kritschenko reaction was studied. After stirring the reaction mixture for 48 h at 323 K in the ethanol/sodium hydroxide system (pH = 10, reaction progress controlled by TLC), the title compound was obtained instead of expected aza­crown ether.

According to the *PASS* program (Filimonov *et al.*, 2014 ▸), which makes a computer prediction of biological activities, the title compound is expected to inhibit gluconate 2-de­hydrogenase activity (85% probability), as well as to be a mucomembranous protector (73%).
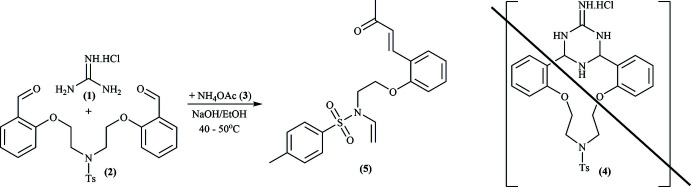


## Structural commentary   

**Figure 1:**
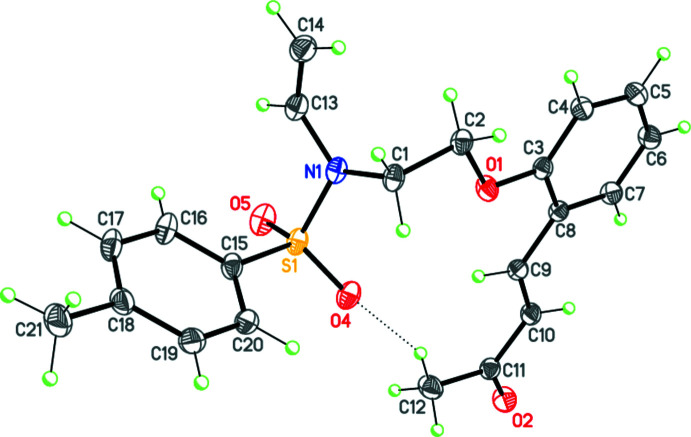
Mol­ecular structure of the title compound with displacement ellipsoids shown at the 50% probability level. The dashed line indicates the intra­molecular CH_2_—H⋯O hydrogen bond.

The title compound is the product of an unexpected transformation starting from 1,5-bis­(1-phen­oxy)-3-aza­pentane. Its mol­ecular structure is presented in Fig. 1 ▸. The mol­ecule contains a tosyl­ated secondary vinyl­amine and a benzalacetone fragment. The benzalacetone fragment adopts a *trans* conformation with respect to the C9=C10 double bond of 1.3432 (14) Å; this is slightly longer than the vinylic C13=C14 bond [1.3278 (16) Å] due to the conjugation with the neighbouring acetyl group. The amine N atom is significantly flattened due to conjugation with a vinyl group, the C1—S1—N1—C13 torsion angle being 28.46 (13)°. The N1—C13 bond distance [1.4138 (13) Å] is slightly shorter than that of a standard C—N single bond in similar compounds (Tskhovrebov *et al.*, 2012 ▸, 2014 ▸, 2018 ▸; Repina *et al.*, 2020 ▸). The mol­ecular structure features an intra­molecular S1=O4⋯H12*B*—C12 hydrogen bond (Table 1 ▸), leading to the formation of an *S*(13) macrocycle in the crystal.

## Supra­molecular features   

**Figure 2:**
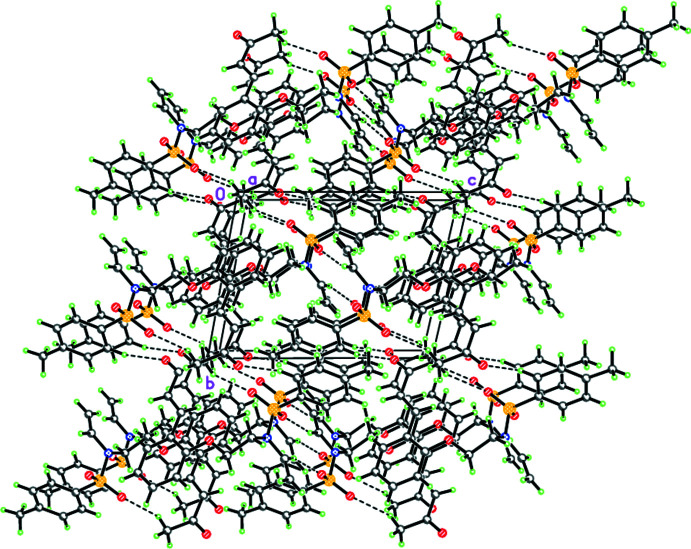
Crystal packing of the title compound illustrating its self-assembly into a hydrogen-bonded framework.

In the crystal, the mol­ecules of the title enamine are linked by pairs of inter­molecular C—H⋯O contacts into chains stretched along the [011] direction (Fig. 2 ▸, Table 1 ▸). A similar supra­molecular motif has previously been observed by our group (Tskhovrebov *et al.*, 2019 ▸; Repina *et al.*, 2020 ▸).

## Database survey   

A search of the Cambridge Structural Database (CSD version 5.41, update of March 2020; Groom *et al.*, 2016 ▸) revealed that this is the first example of a structurally characterized compound that contains an *N*-tosyl­ated vinyl­amine fragment. At the same time, the CSD revealed the existence of some examples of structurally similar vinyl ketones, *viz*. 1-(4-chloro­phen­yl)-3-(2,4,5-tri­meth­oxy­phen­yl)prop-2-en-1-one (Teh *et al.*, 2006 ▸), (2*E*)-1-(pyridin-2-yl)-3-(2,4,6-tri­meth­oxy­phen­yl)prop-2-en-1-one (Fun *et al.*, 2011 ▸), (2*E*)-1-(pyridin-2-yl)-3-(2,4,5-tri­meth­oxy­phen­yl)prop-2-en-1-one (Chantra­prom­ma *et al.*, 2013 ▸) and (1*E*,4*Z*,6*E*)-5-hy­droxy-1,7-bis­(2-meth­oxy­phen­yl)-1,4,6- hepta­trien-3-one (Zhao *et al.*, 2011 ▸).

## Synthesis and crystallization   

Equimolar amounts of 1,5-bis­(1-phen­oxy)-3-aza­pentane (0.34 mmol, 0.16 g) and guanidine hydro­chloride (0.34 mmol, 0.03 g) were stirred in an ethanol/sodium hydroxide mixture at 313–323 K in the presence of ammonium acetate (3.38 mmol, 0.26 g). The reaction was monitored by TLC and completed after 48 h. The reaction mixture was allowed to cool to room temperature (298 K). Then, the product was extracted with di­chloro­methane (3 × 30 ml) and dried with Na_2_SO_4_. The solvent was evaporated under reduced pressure, the residue was purified by column chromatography and recrystallized from di­chloro­methane to obtain single crystals of the unexpected enamine. *T*_mlt_ = 403–404 K; *R*_f_ = 0.53, eluent: hexa­ne/ethyl­acetate = 2:1, silufol. ^1^H NMR (CDCl_3_, 500 MHz, 300 K), δ, ppm: 9.79–9.81 (*m*, 1H, –C_6_H_4_—CH=CH–), 7.76–7.81 (*m*, 3H), 7.53 (*d*, 1H, *J* = 7.5 Hz), 7.29–7.34 (*m*, 3H), 6.99 (*t*, 1H, *J* = 7.5 Hz), 6.82 (*d*, 1H, *J* = 8.5 Hz), 6.70 (*d*, 1H, *J* = 16.5 Hz), 4.10 (*t*, 2H, *J* = 5.5 Hz, –O—CH_2_–), 3.41–3.44 (*m*, 2H, –N—CH_2_–), 2.41 (*s*, 3H, CH_3_—C_6_H_4_–); 2.36 (*s*, 3H, CH_3_—C=O), 2.20 (*d*, 2H, *J* = 3 Hz).

## Refinement   

Crystal data, data collection and structure refinement details are summarized in Table 2 ▸. The hydrogen atoms were placed in calculated positions with C—H = 0.95–0.99 Å and refined as riding with fixed isotropic displacement parameters [*U*_iso_(H) = 1.2–1.5*U*_eq_(C)].

## Supplementary Material

Crystal structure: contains datablock(s) I. DOI: 10.1107/S2056989020015194/yk2141sup1.cif

Structure factors: contains datablock(s) I. DOI: 10.1107/S2056989020015194/yk2141Isup2.hkl

1H NMR Spetrum of unexpected product. DOI: 10.1107/S2056989020015194/yk2141sup3.pdf

Supporting information file. DOI: 10.1107/S2056989020015194/yk2141Isup4.cml

CCDC reference: 2044436

Additional supporting information:  crystallographic information; 3D view; checkCIF report

## Figures and Tables

**Table 1:** Hydrogen-bond geometry (Å, °)

*D*—H⋯*A*	*D*—H	H⋯*A*	*D*⋯*A*	*D*—H⋯*A*
C12—H12*B*⋯O4	0.98	2.61	3.5193 (14)	155
C13—H13⋯O5^i^	0.95	2.35	3.2307 (13)	154
C20—H20⋯O2^ii^	0.95	2.42	3.3070 (14)	156

Symmetry codes: (i) 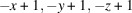; (ii) 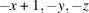.

**Table 2:** Experimental details

Crystal data	
Chemical formula	C_21_H_23_NO_4_S
*M*_r_	385.46
Crystal system, space group	Triclinic, *P* 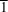
Temperature (K)	100
*a*, *b*, *c* (Å)	8.9428 (4), 9.5089 (4), 12.1090 (5)
α, β, γ (°)	100.395 (1), 91.739 (1), 108.970 (1)
*V* (Å^3^)	953.40 (7)
*Z*	2
Radiation type	Mo *K*α
μ (mm^−1^)	0.20
Crystal size (mm)	0.30 × 0.25 × 0.20

Data collection	
Diffractometer	Bruker D8 QUEST PHOTON-III CCD
Absorption correction	Multi-scan (*SADABS*; Bruker, 2018 ▸)
*T*_min_, *T*_max_	0.936, 0.954
No. of measured, independent and observed [*I* > 2σ(*I*)] reflections	22783, 6917, 6035
*R*_int_	0.025
(sin θ/λ)_max_ (Å^−1^)	0.758

Refinement	
*R*[*F* ^2^ > 2σ(*F* ^2^)], *wR*(*F* ^2^), *S*	0.041, 0.114, 1.03
No. of reflections	6917
No. of parameters	246
H-atom treatment	H-atom parameters constrained
Δρ_max_, Δρ_min_ (e Å^−3^)	0.64, −0.59

Computer programs: *APEX3* and *SAINT* (Bruker, 2018 ▸), *SHELXT* (Sheldrick, 2015*a* ▸), *SHELXL* (Sheldrick, 2015*b* ▸) and *SHELXTL* (Sheldrick, 2015*b* ▸).

## supplementary crystallographic information

### Crystal data

**Table:**

C_21_H_23_NO_4_S	*Z* = 2
*M**_r_* = 385.46	*F*(000) = 408
Triclinic, *P*1	*D*_x_ = 1.343 Mg m^−^^3^
*a* = 8.9428 (4) Å	Mo *K*α radiation, λ = 0.71073 Å
*b* = 9.5089 (4) Å	Cell parameters from 9902 reflections
*c* = 12.1090 (5) Å	θ = 2.8–32.6°
α = 100.395 (1)°	µ = 0.20 mm^−^^1^
β = 91.739 (1)°	*T* = 100 K
γ = 108.970 (1)°	Prism, colourless
*V* = 953.40 (7) Å^3^	0.30 × 0.25 × 0.20 mm

### Data collection

**Table:**

Bruker D8 QUEST PHOTON-III CCD diffractometer	6035 reflections with *I* > 2σ(*I*)
φ and ω scans	*R*_int_ = 0.025
Absorption correction: multi-scan (SADABS; Bruker, 2018)	θ_max_ = 32.6°, θ_min_ = 2.6°
*T*_min_ = 0.936, *T*_max_ = 0.954	*h* = −13→13
22783 measured reflections	*k* = −14→14
6917 independent reflections	*l* = −18→18

### Refinement

**Table:**

Refinement on *F*^2^	Primary atom site location: difference Fourier map
Least-squares matrix: full	Secondary atom site location: difference Fourier map
*R*[*F*^2^ > 2σ(*F*^2^)] = 0.041	Hydrogen site location: inferred from neighbouring sites
*wR*(*F*^2^) = 0.114	H-atom parameters constrained
*S* = 1.03	*w* = 1/[σ^2^(*F*_o_^2^) + (0.0563*P*)^2^ + 0.3812*P*] where *P* = (*F*_o_^2^ + 2*F*_c_^2^)/3
6917 reflections	(Δ/σ)_max_ = 0.001
246 parameters	Δρ_max_ = 0.64 e Å^−^^3^
0 restraints	Δρ_min_ = −0.59 e Å^−^^3^

### Special details

**Table:**

Geometry. All esds (except the esd in the dihedral angle between two l.s. planes) are estimated using the full covariance matrix. The cell esds are taken into account individually in the estimation of esds in distances, angles and torsion angles; correlations between esds in cell parameters are only used when they are defined by crystal symmetry. An approximate (isotropic) treatment of cell esds is used for estimating esds involving l.s. planes.

### Fractional atomic coordinates and isotropic or equivalent isotropic displacement parameters (Å^2^)

**Table:**

	*x*	*y*	*z*	*U*_iso_*/*U*_eq_	
S1	0.58516 (3)	0.27374 (3)	0.35034 (2)	0.01970 (7)	
O1	0.77929 (9)	0.44036 (9)	0.10904 (6)	0.02095 (15)	
O2	0.16458 (10)	0.00224 (10)	−0.17227 (7)	0.02659 (17)	
O4	0.56420 (10)	0.17414 (10)	0.24326 (6)	0.02473 (16)	
O5	0.45799 (10)	0.32396 (11)	0.38963 (7)	0.02719 (17)	
N1	0.73561 (10)	0.42912 (10)	0.34653 (7)	0.01924 (16)	
C1	0.87112 (12)	0.41498 (12)	0.28577 (8)	0.01923 (17)	
H1A	0.848514	0.307849	0.248005	0.023*	
H1B	0.966656	0.445383	0.339898	0.023*	
C2	0.90243 (12)	0.51458 (12)	0.19871 (8)	0.01925 (17)	
H2A	0.899686	0.616788	0.231586	0.023*	
H2B	1.007800	0.525374	0.170965	0.023*	
C3	0.77421 (11)	0.50949 (11)	0.02042 (8)	0.01681 (16)	
C4	0.88959 (12)	0.64411 (12)	0.00796 (9)	0.01946 (18)	
H4	0.977426	0.693521	0.063383	0.023*	
C5	0.87526 (13)	0.70560 (12)	−0.08612 (9)	0.02104 (18)	
H5	0.954362	0.796595	−0.095238	0.025*	
C6	0.74632 (13)	0.63502 (12)	−0.16670 (9)	0.02164 (19)	
H6	0.736959	0.677639	−0.230659	0.026*	
C7	0.63121 (12)	0.50205 (12)	−0.15349 (8)	0.01962 (18)	
H7	0.542447	0.455152	−0.208445	0.024*	
C8	0.64272 (11)	0.43520 (11)	−0.06096 (8)	0.01636 (16)	
C9	0.52332 (12)	0.29496 (11)	−0.04462 (8)	0.01731 (17)	
H9	0.533444	0.264425	0.024932	0.021*	
C10	0.40059 (12)	0.20569 (12)	−0.11953 (8)	0.01997 (18)	
H10	0.392242	0.234084	−0.190162	0.024*	
C11	0.27812 (12)	0.06751 (12)	−0.10098 (8)	0.01966 (18)	
C12	0.29034 (15)	0.00462 (13)	0.00273 (10)	0.0269 (2)	
H12A	0.190753	−0.012441	0.038377	0.040*	
H12B	0.378057	0.076981	0.055774	0.040*	
H12C	0.310181	−0.091767	−0.018228	0.040*	
C13	0.76139 (13)	0.55211 (12)	0.43844 (9)	0.02207 (19)	
H13	0.671419	0.559367	0.475690	0.026*	
C14	0.90037 (15)	0.65837 (13)	0.47723 (10)	0.0261 (2)	
H14A	0.993489	0.655552	0.442516	0.031*	
H14B	0.906687	0.737205	0.539687	0.031*	
C15	0.64829 (12)	0.19288 (12)	0.45430 (8)	0.01979 (18)	
C16	0.64349 (13)	0.25213 (14)	0.56761 (9)	0.0244 (2)	
H16	0.599326	0.330349	0.588562	0.029*	
C17	0.70433 (14)	0.19488 (14)	0.64927 (9)	0.0253 (2)	
H17	0.701353	0.234515	0.726703	0.030*	
C18	0.76971 (13)	0.08036 (12)	0.61987 (9)	0.0235 (2)	
C19	0.77088 (15)	0.02169 (13)	0.50596 (10)	0.0257 (2)	
H19	0.813180	−0.057844	0.484864	0.031*	
C20	0.71119 (14)	0.07763 (12)	0.42281 (9)	0.02283 (19)	
H20	0.713379	0.037533	0.345344	0.027*	
C21	0.84096 (17)	0.02345 (15)	0.70901 (10)	0.0315 (3)	
H21A	0.803334	−0.087790	0.691599	0.047*	
H21B	0.809001	0.058972	0.782650	0.047*	
H21C	0.957032	0.062056	0.710939	0.047*	

### Atomic displacement parameters (Å^2^)

**Table:**

	*U*^11^	*U*^22^	*U*^33^	*U*^12^	*U*^13^	*U*^23^
S1	0.01717 (11)	0.02807 (13)	0.01380 (11)	0.00833 (9)	−0.00154 (8)	0.00327 (8)
O1	0.0210 (3)	0.0247 (3)	0.0154 (3)	0.0037 (3)	−0.0028 (3)	0.0078 (3)
O2	0.0215 (4)	0.0302 (4)	0.0219 (4)	0.0013 (3)	−0.0035 (3)	0.0047 (3)
O4	0.0261 (4)	0.0307 (4)	0.0148 (3)	0.0086 (3)	−0.0042 (3)	0.0010 (3)
O5	0.0184 (3)	0.0418 (5)	0.0228 (4)	0.0135 (3)	−0.0004 (3)	0.0041 (3)
N1	0.0204 (4)	0.0247 (4)	0.0148 (3)	0.0103 (3)	0.0020 (3)	0.0040 (3)
C1	0.0202 (4)	0.0253 (4)	0.0153 (4)	0.0112 (4)	0.0017 (3)	0.0055 (3)
C2	0.0184 (4)	0.0241 (4)	0.0150 (4)	0.0070 (3)	−0.0009 (3)	0.0042 (3)
C3	0.0183 (4)	0.0193 (4)	0.0141 (4)	0.0078 (3)	0.0012 (3)	0.0039 (3)
C4	0.0179 (4)	0.0210 (4)	0.0188 (4)	0.0053 (3)	0.0005 (3)	0.0048 (3)
C5	0.0212 (4)	0.0204 (4)	0.0225 (4)	0.0066 (3)	0.0028 (4)	0.0075 (4)
C6	0.0246 (5)	0.0224 (4)	0.0203 (4)	0.0090 (4)	0.0006 (4)	0.0084 (4)
C7	0.0217 (4)	0.0211 (4)	0.0173 (4)	0.0085 (3)	−0.0013 (3)	0.0052 (3)
C8	0.0182 (4)	0.0171 (4)	0.0149 (4)	0.0076 (3)	0.0009 (3)	0.0030 (3)
C9	0.0189 (4)	0.0175 (4)	0.0166 (4)	0.0075 (3)	0.0009 (3)	0.0036 (3)
C10	0.0210 (4)	0.0214 (4)	0.0161 (4)	0.0051 (3)	−0.0001 (3)	0.0043 (3)
C11	0.0195 (4)	0.0211 (4)	0.0172 (4)	0.0059 (3)	0.0006 (3)	0.0029 (3)
C12	0.0305 (5)	0.0237 (5)	0.0228 (5)	0.0033 (4)	−0.0039 (4)	0.0079 (4)
C13	0.0263 (5)	0.0258 (5)	0.0176 (4)	0.0135 (4)	0.0024 (4)	0.0042 (4)
C14	0.0310 (5)	0.0255 (5)	0.0220 (5)	0.0107 (4)	0.0006 (4)	0.0036 (4)
C15	0.0177 (4)	0.0251 (4)	0.0145 (4)	0.0042 (3)	−0.0007 (3)	0.0047 (3)
C16	0.0238 (5)	0.0358 (6)	0.0151 (4)	0.0124 (4)	0.0021 (3)	0.0044 (4)
C17	0.0246 (5)	0.0354 (6)	0.0141 (4)	0.0071 (4)	0.0010 (3)	0.0060 (4)
C18	0.0254 (5)	0.0222 (4)	0.0186 (4)	0.0007 (4)	−0.0025 (4)	0.0076 (4)
C19	0.0352 (6)	0.0203 (4)	0.0203 (4)	0.0079 (4)	−0.0023 (4)	0.0044 (4)
C20	0.0293 (5)	0.0205 (4)	0.0158 (4)	0.0053 (4)	−0.0019 (4)	0.0029 (3)
C21	0.0394 (6)	0.0303 (6)	0.0234 (5)	0.0072 (5)	−0.0050 (5)	0.0120 (4)

### Geometric parameters (Å, º)

**Table:**

S1—O4	1.4284 (8)	C9—H9	0.9500
S1—O5	1.4323 (8)	C10—C11	1.4705 (14)
S1—N1	1.6527 (10)	C10—H10	0.9500
S1—C15	1.7559 (10)	C11—C12	1.5006 (15)
O1—C3	1.3625 (12)	C12—H12A	0.9800
O1—C2	1.4267 (12)	C12—H12B	0.9800
O2—C11	1.2268 (12)	C12—H12C	0.9800
N1—C13	1.4138 (13)	C13—C14	1.3278 (16)
N1—C1	1.4671 (13)	C13—H13	0.9500
C1—C2	1.5142 (14)	C14—H14A	0.9500
C1—H1A	0.9900	C14—H14B	0.9500
C1—H1B	0.9900	C15—C20	1.3878 (16)
C2—H2A	0.9900	C15—C16	1.3936 (14)
C2—H2B	0.9900	C16—C17	1.3871 (16)
C3—C4	1.3952 (14)	C16—H16	0.9500
C3—C8	1.4111 (13)	C17—C18	1.3933 (17)
C4—C5	1.3915 (14)	C17—H17	0.9500
C4—H4	0.9500	C18—C19	1.3931 (16)
C5—C6	1.3872 (15)	C18—C21	1.5024 (16)
C5—H5	0.9500	C19—C20	1.3881 (15)
C6—C7	1.3859 (15)	C19—H19	0.9500
C6—H6	0.9500	C20—H20	0.9500
C7—C8	1.4009 (13)	C21—H21A	0.9800
C7—H7	0.9500	C21—H21B	0.9800
C8—C9	1.4628 (13)	C21—H21C	0.9800
C9—C10	1.3432 (14)		
			
O4—S1—O5	120.10 (5)	C9—C10—C11	125.23 (9)
O4—S1—N1	107.17 (5)	C9—C10—H10	117.4
O5—S1—N1	106.02 (5)	C11—C10—H10	117.4
O4—S1—C15	109.04 (5)	O2—C11—C10	119.11 (9)
O5—S1—C15	108.29 (5)	O2—C11—C12	119.63 (10)
N1—S1—C15	105.22 (5)	C10—C11—C12	121.26 (9)
C3—O1—C2	118.61 (8)	C11—C12—H12A	109.5
C13—N1—C1	118.85 (9)	C11—C12—H12B	109.5
C13—N1—S1	116.22 (7)	H12A—C12—H12B	109.5
C1—N1—S1	118.83 (7)	C11—C12—H12C	109.5
N1—C1—C2	110.33 (8)	H12A—C12—H12C	109.5
N1—C1—H1A	109.6	H12B—C12—H12C	109.5
C2—C1—H1A	109.6	C14—C13—N1	125.70 (10)
N1—C1—H1B	109.6	C14—C13—H13	117.2
C2—C1—H1B	109.6	N1—C13—H13	117.2
H1A—C1—H1B	108.1	C13—C14—H14A	120.0
O1—C2—C1	106.03 (8)	C13—C14—H14B	120.0
O1—C2—H2A	110.5	H14A—C14—H14B	120.0
C1—C2—H2A	110.5	C20—C15—C16	121.03 (10)
O1—C2—H2B	110.5	C20—C15—S1	119.58 (8)
C1—C2—H2B	110.5	C16—C15—S1	119.27 (9)
H2A—C2—H2B	108.7	C17—C16—C15	118.83 (11)
O1—C3—C4	123.68 (9)	C17—C16—H16	120.6
O1—C3—C8	115.50 (8)	C15—C16—H16	120.6
C4—C3—C8	120.82 (9)	C16—C17—C18	121.25 (10)
C5—C4—C3	119.62 (9)	C16—C17—H17	119.4
C5—C4—H4	120.2	C18—C17—H17	119.4
C3—C4—H4	120.2	C19—C18—C17	118.70 (10)
C6—C5—C4	120.49 (9)	C19—C18—C21	120.51 (11)
C6—C5—H5	119.8	C17—C18—C21	120.78 (10)
C4—C5—H5	119.8	C20—C19—C18	121.03 (11)
C7—C6—C5	119.71 (9)	C20—C19—H19	119.5
C7—C6—H6	120.1	C18—C19—H19	119.5
C5—C6—H6	120.1	C15—C20—C19	119.14 (10)
C6—C7—C8	121.52 (9)	C15—C20—H20	120.4
C6—C7—H7	119.2	C19—C20—H20	120.4
C8—C7—H7	119.2	C18—C21—H21A	109.5
C7—C8—C3	117.82 (9)	C18—C21—H21B	109.5
C7—C8—C9	122.84 (9)	H21A—C21—H21B	109.5
C3—C8—C9	119.32 (8)	C18—C21—H21C	109.5
C10—C9—C8	125.58 (9)	H21A—C21—H21C	109.5
C10—C9—H9	117.2	H21B—C21—H21C	109.5
C8—C9—H9	117.2		
			
O4—S1—N1—C13	170.89 (7)	C7—C8—C9—C10	7.68 (16)
O5—S1—N1—C13	41.49 (8)	C3—C8—C9—C10	−173.69 (10)
C15—S1—N1—C13	−73.13 (8)	C8—C9—C10—C11	−177.90 (9)
O4—S1—N1—C1	−36.83 (9)	C9—C10—C11—O2	174.10 (11)
O5—S1—N1—C1	−166.24 (7)	C9—C10—C11—C12	−6.41 (16)
C15—S1—N1—C1	79.15 (8)	C1—N1—C13—C14	−0.80 (16)
C13—N1—C1—C2	−83.12 (11)	S1—N1—C13—C14	151.47 (10)
S1—N1—C1—C2	125.34 (8)	O4—S1—C15—C20	17.84 (10)
C3—O1—C2—C1	177.09 (8)	O5—S1—C15—C20	150.13 (9)
N1—C1—C2—O1	−74.11 (10)	N1—S1—C15—C20	−96.84 (9)
C2—O1—C3—C4	4.89 (14)	O4—S1—C15—C16	−166.14 (9)
C2—O1—C3—C8	−175.48 (8)	O5—S1—C15—C16	−33.85 (10)
O1—C3—C4—C5	179.33 (9)	N1—S1—C15—C16	79.18 (9)
C8—C3—C4—C5	−0.28 (15)	C20—C15—C16—C17	0.61 (17)
C3—C4—C5—C6	0.77 (16)	S1—C15—C16—C17	−175.35 (9)
C4—C5—C6—C7	−0.18 (16)	C15—C16—C17—C18	0.11 (17)
C5—C6—C7—C8	−0.92 (16)	C16—C17—C18—C19	−1.03 (17)
C6—C7—C8—C3	1.37 (15)	C16—C17—C18—C21	177.61 (11)
C6—C7—C8—C9	−179.99 (9)	C17—C18—C19—C20	1.27 (17)
O1—C3—C8—C7	179.59 (9)	C21—C18—C19—C20	−177.38 (11)
C4—C3—C8—C7	−0.76 (14)	C16—C15—C20—C19	−0.38 (17)
O1—C3—C8—C9	0.90 (13)	S1—C15—C20—C19	175.57 (9)
C4—C3—C8—C9	−179.46 (9)	C18—C19—C20—C15	−0.58 (17)

### Hydrogen-bond geometry (Å, º)

**Table:**

*D*—H···*A*	*D*—H	H···*A*	*D*···*A*	*D*—H···*A*
C12—H12*B*···O4	0.98	2.61	3.5193 (14)	155
C13—H13···O5^i^	0.95	2.35	3.2307 (13)	154
C20—H20···O2^ii^	0.95	2.42	3.3070 (14)	156

Symmetry codes: (i) −*x*+1, −*y*+1, −*z*+1; (ii) −*x*+1, −*y*, −*z*.
