# Supplementary material for: Unexpected synthesis and crystal structure of N-{2-[2-(2-acetyl­ethen­yl)phen­oxy]eth­yl}-N-ethenyl-4-methyl­benzene­sulfonamide
Source: Acta Crystallogr E Crystallogr Commun. 2020 Nov 20;76(Pt 12):1851–3. doi: 10.1107/S2056989020015194 (PMC7784650; doi:10.1107/S2056989020015194)

LG09-CDCl3-1H

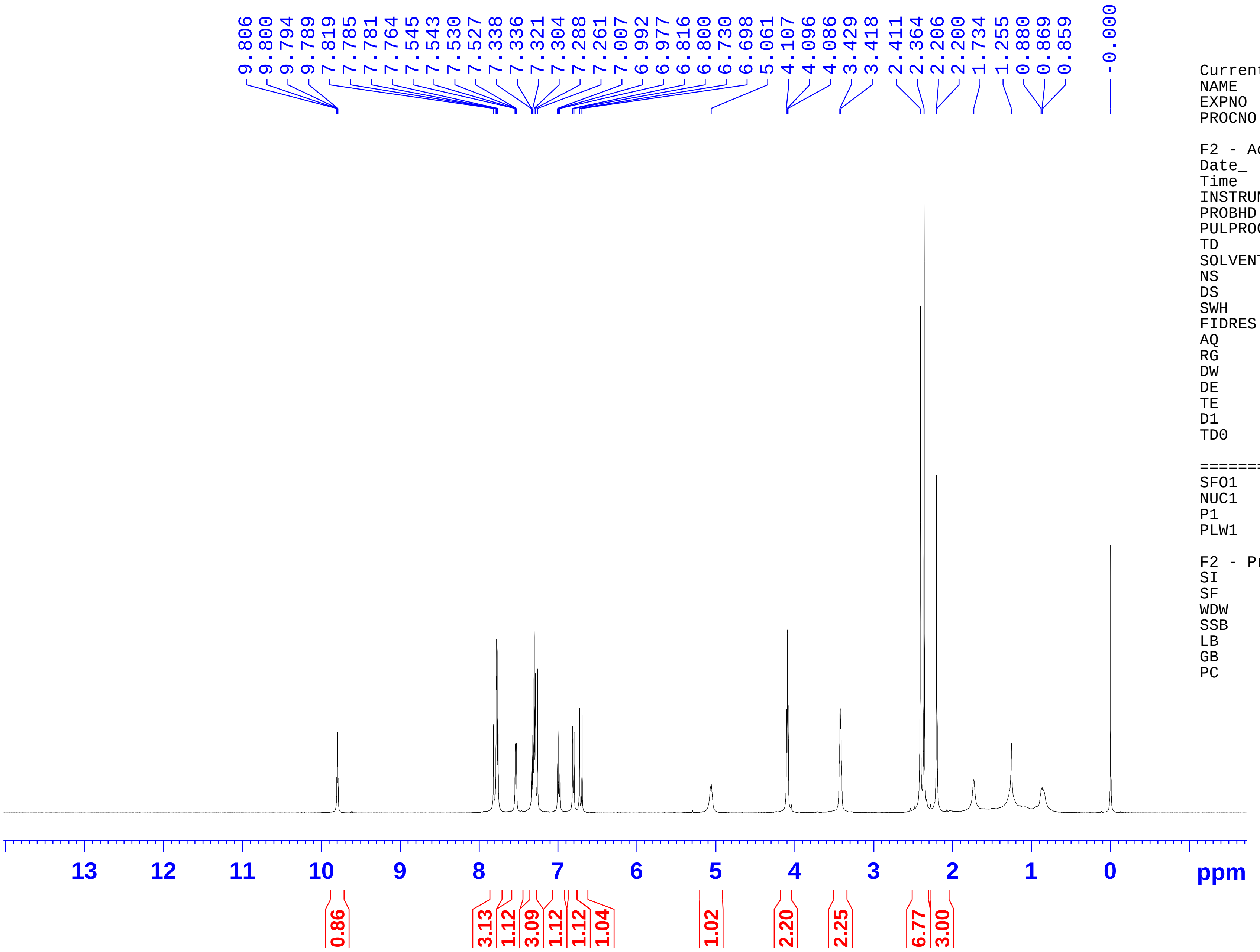


```
Current Data Parameters
NAME              DAT_LG09
EXPNO                   10
PROCNO                   1

F2 - Acquisition Parameters
Date_             20200924
Time                 17.33
INSTRUM              spect
PROBHD    5 mm PABBO BB/
PULPROG               zg30
TD                   65536
SOLVENT              CDCl3
NS                      16
DS                       2
SWH             10000.000 Hz
FIDRES           0.152588 Hz
AQ              3.2767999 sec
RG                  157.35
DW                  50.000 usec
DE                    6.50 usec
TE                   301.9 K
D1             1.00000000 sec
TD0                      1

======== CHANNEL f1 ========
SFO1          500.2420892 MHz
NUC1                    1H
P1                   10.20 usec
PLW1           22.00000000 W

F2 - Processing parameters
SI                   65536
SF            500.2390125 MHz
WDW                     EM
SSB         0
LB                    0.30 Hz
GB          0
PC                    1.00
```

LG09-CDCl3-1H

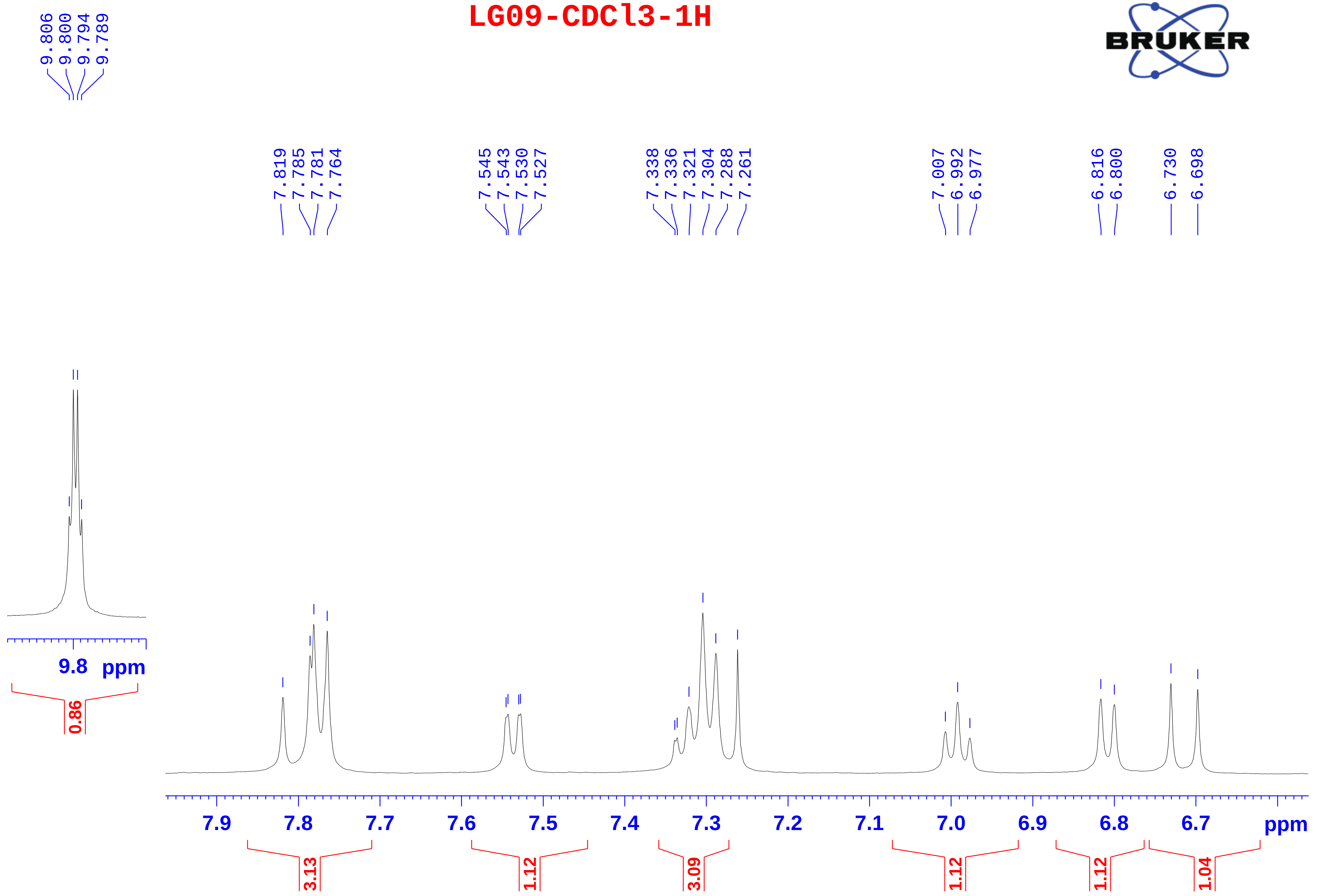

LG09-CDCl3-1H

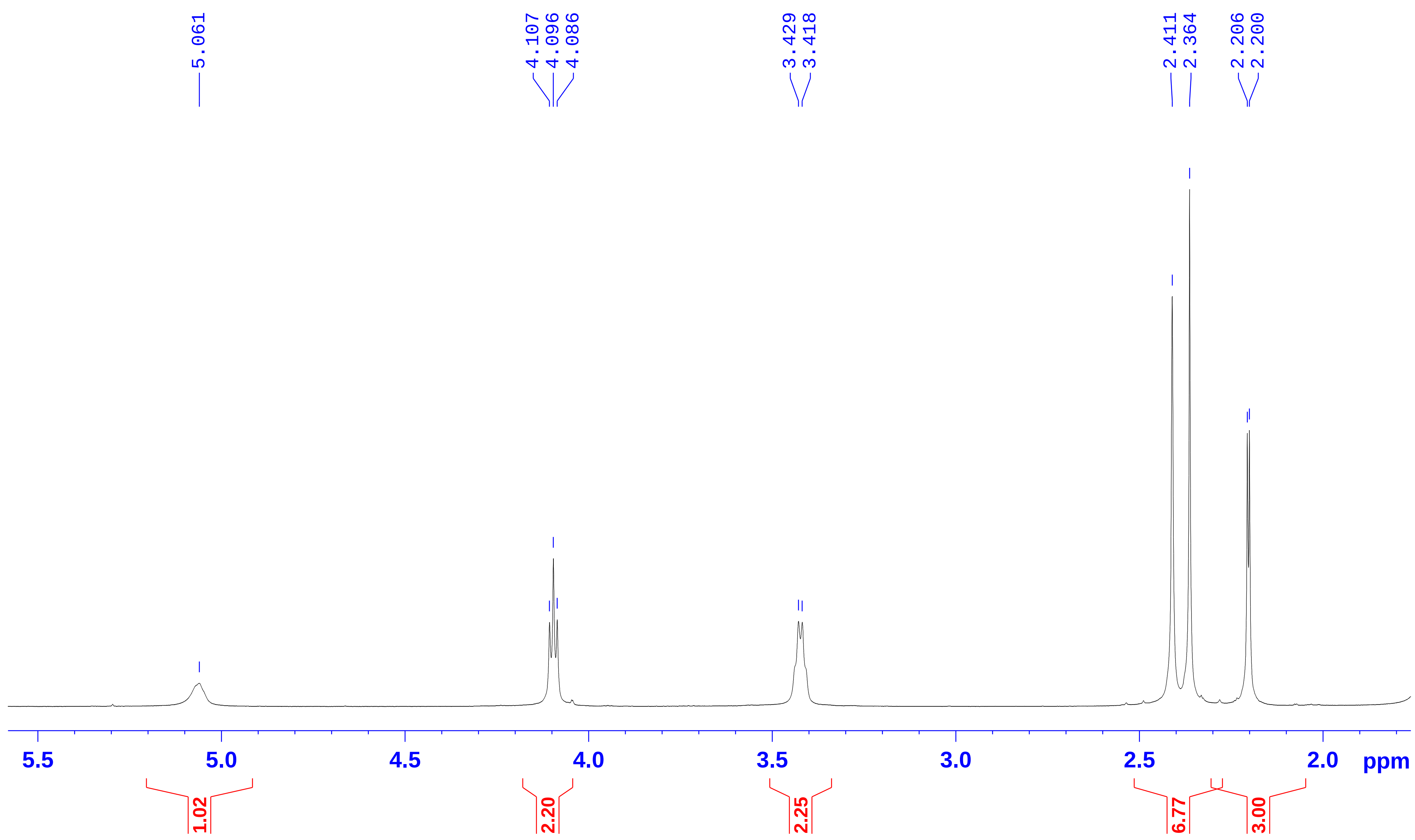

Supplement: Supplementary file 3 [file e-76-01851-sup3.pdf]
